# Microfluidic Mixing and Analog On-Chip Concentration Control Using Fluidic Dielectrophoresis

**DOI:** 10.3390/mi7110214

**Published:** 2016-11-23

**Authors:** Nicholas Mavrogiannis, Mitchell Desmond, Kenny Ling, Xiaotong Fu, Zachary Gagnon

**Affiliations:** Department of Chemical and Biomolecular Engineering, Johns Hopkins University, Maryland Hall 220A, Baltimore, MD 21218, USA; nmavrog1@jhu.edu (N.M.); mdesmond42@gmail.com (M.D.); kling2@jhu.edu (K.L.); xfu7@jhu.edu (X.F.)

**Keywords:** mixing, fluidic dielectrophoresis, microfluidics, laminar flow, chemical gradient

## Abstract

Microfluidic platforms capable of complex on-chip processing and liquid handling enable a wide variety of sensing, cellular, and material-related applications across a spectrum of disciplines in engineering and biology. However, there is a general lack of available active microscale mixing methods capable of dynamically controlling on-chip solute concentrations in real-time. Hence, multiple microfluidic fluid handling steps are often needed for applications that require buffers at varying on-chip concentrations. Here, we present a novel electrokinetic method for actively mixing laminar fluids and controlling on-chip concentrations in microfluidic channels using fluidic dielectrophoresis. Using a microfluidic channel junction, we co-flow three electrolyte streams side-by-side so that two outer conductive streams enclose a low conductive central stream. The tri-laminar flow is driven through an array of electrodes where the outer streams are electrokinetically deflected and forced to mix with the central flow field. This newly mixed central flow is then sent continuously downstream to serve as a concentration boundary condition for a microfluidic gradient chamber. We demonstrate that by actively mixing the upstream fluids, a variable concentration gradient can be formed dynamically downstream with single a fixed inlet concentration. This novel mixing approach offers a useful method for producing variable on-chip concentrations from a single inlet source.

## 1. Introduction

Microfluidics is emerging as a fundamental engineering science for development of lab-on-a-chip (LOC) and point-of-care diagnostic platforms. A primary objective for LOC design is to miniaturize conventional chemical and biological analysis processes so they can function without human intervention on-chip. The benefits of using microfluidics to support LOC technology are well known and include shorter analysis times, reduced sample volume, and significantly reduced manufacturing and operation costs. One challenge in designing miniaturized LOC processes is reagent mixing. Common laboratory tasks such as protein immunoassays [1,2], DNA hybridization [3,4], and cell culture [5] all require well-controlled mixing. Mixing at low Reynolds number [6], however, can be challenging since inertial contributions to fluid motion are small and macromolecules such as protein and DNA often have low diffusion coefficients. Additionally, microfluidic mixing is often binary in nature. A fluid, for example, with an initial solute concentration is often combined and mixed together with another fluid to create a new, but fixed, solute concentration that is different from the original solution. The ability to dynamically control solute concentration during this process is challenging, and microfluidic processes often require significant off-chip sample preparation and multiple mixing steps on-chip to create mixed solutions at desired on-chip concentrations.

Microfluidic mixing can be accomplished either passively with secondary flow fields or actively using externally applied fluid forces. Because the Reynolds number is often well below one, these passive methods create mixing by stretching the interface between two liquid streams to decrease the distance over which diffusion occurs. Passive mixing approaches focus on increasing mixing fluid contact area or contact time to promote enhanced diffusion between flows. One popular passive mixing method uses microfluidic lamination, where multiple streams a forced to split and then later gather as a multitude of smaller streams in order to increase the contact area between the flows [7]. While lamination can provide fast mixing times and shorter mixing lengths [8], it typically requires complex fabrication methods and a complicated network of microchannels. Other passive mixers rely on the use of patterned surface chemistry [9], 3-D serpentine structures [10,11] and baffles or obstacles to create secondary flows for mixing [12,13,14,15,16]. While effective, passive methods create disperse samples, and the final on-chip solute concentration is diluted as it spreads out longitudinally into neighboring buffer during the mixing process. Additionally, while passive mixing devices can typically mix within 55–300 ms, many devices result in lower mixing efficiency, and therefore require a longer mixing to achieve optimal mixing. 

An alternative to passive mixing is active mixing, where fluid streams are combined with the use of an external driving force [17,18]. Common active methods include mixing by peristaltic pumps [19], magnetic particles [20,21,22] or surface acoustic waves [23,24]. Because active mixing can often produce flow fields with larger fluid velocities than passive methods, active mixing methods generally have shorter on-chip mixing dimensions and mixing times. One way to actively drive fluid mixing is to use electrokinetics. In this approach, an electrical field is introduced into a microchannel. The field interacts with regions of non-neutral space charge to drive fluid motion. These electrokinetic flows can then be harnessed to drive the mixing of two of more fluidic streams.

While many microfluidic mixing methods exist, they are all based on the principle of binary dilution, where two or more flow volumes are combined to produce a larger volume at a fixed diluted concentration. While both active and passive mixing techniques have been effective in combating the lack of inertial influence at low Reynolds number, it is currently very difficult to control on-chip concentration in real-time during mixing. Here, we present a novel electrokinetic mixing approach for dynamically tuning and controlling the on-chip solute concentration of fluid flows. Mixing is accomplished using an externally applied alternating current (AC) electric field dropped across a microchannel with three co-flowing liquid streams. Two outer streams contain a solute to be mixed with the central stream, while the inner central stream flows downstream to a chemical gradient generator. The laminar flow field is electrokinetically driven across the microchannel by the electric field and the magnitude of fluid motion can be controlled by varying the frequency and electric field strength. We show that on-chip solute concentration in the central flow stream can be dynamically controlled and demonstrate usefulness of this method by controlling the concentration in a downstream microfluidic gradient generator. 

## 2. Experimental Section

**Figure 1:**
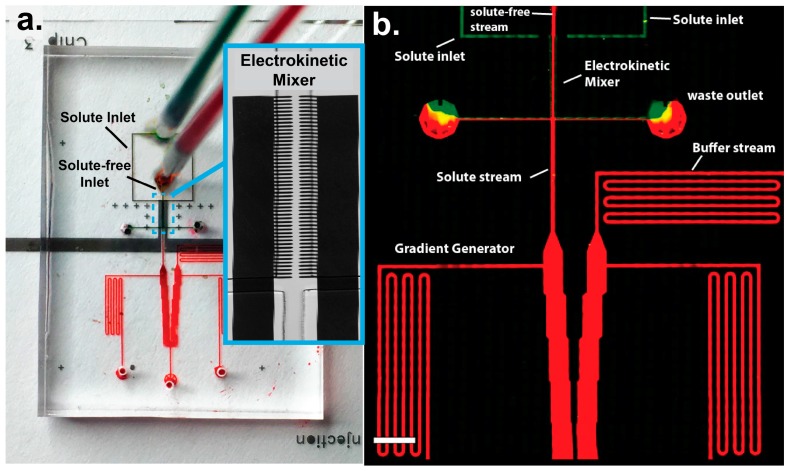
(**a**) Microfludic device with embedded electrodes. The solute and solute-free streams are delivered to the top and bottom inlets respectively. The electrokinetic mixer, boxed in blue, is shown in a magnified brightfield image; (**b**) Montage of the dual-stage microfluidic device. The upstream stage consists of an electrokinetic mixer which is fed continuously downstream to a gradient generator. An electric field is used to inject two solute streams into a central solute-free stream. This stream then serves as an input to the downstream gradient generator. Scale bar, 100 μm.

Electrokinetic mixing experiments were performed using a two-stage microfluidic device (Figure 1). An upstream stage was used to electrokinetically mix multiple laminar streams into a single stream at a desired concentration. This mixed stream was then continuously fed into a downstream stage where it was used to create a stable microfluidic gradient. The overall two-stage design offered a means to assess fluidic mixing by our electrokinetic approach, and to demonstrate dynamic on-chip concentration control.

**Figure 2:**
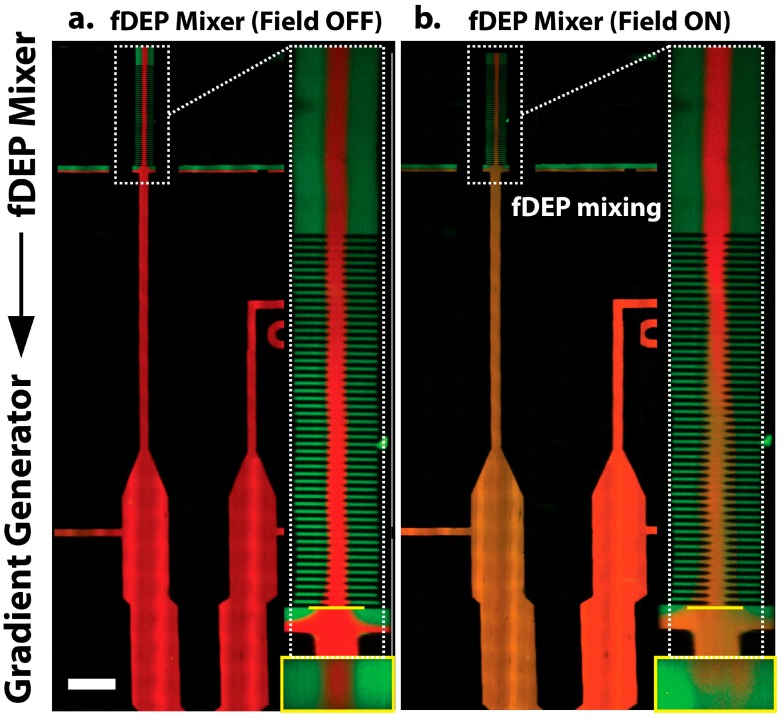
(**a**) Left: The microfluidic device consists of two stages: an upstream mixing stage with embedded electrodes and a downstream gradient chamber. The microchannel network is designed such that when no alternating current (AC) field is applied, only the low conductive central red stream flows to the gradient chamber. Right: A magnified micrograph of the mixing section. A yellow line depicts the spatial location where a 3D cross-sectional image was capture, shown under the zoomed in image; (**b**) When an AC electric field was applied, mixing occurred and influence the solute concentration supplied to the gradient chamber. The mixing section shows that while the red stream enters the mixing zone and exits with a slight orange hue due to mixing with the two outer streams. Scale bar, 200 μm.

The upstream electrokinetic mixing was performed using a combination of laminar and field-induced electrokinetic flow. An array of point electrodes drive fluid into motion by electrokinetic flow using fluidic dielectrophoresis (fDEP). In fDEP, laminar liquid interfaces are polarized and driven into motion by an externally applied AC electric field [25]. To produce fDEP mixing, three different fluid streams were introduced into a main flow channel using an external constant pressure source and made to flow side-by-side in a main flow channel. The electrical properties of the two outer streams were adjusted such that they had a higher conductivity and lower dielectric constant than the inner fluid stream. Therefore, two electrical liquid interfaces were produced that could then be polarized and driven into motion with an electric field. The fluidic channel network was designed such that the two outer streams were driven into waste outlets, while the central stream remained in the device and was sent continuously downstream to a gradient chamber. Therefore, only a “clean” central fluid is sent downstream, while the two outer, high conductive green streams are forced to flow to outlet waste streams. In this work, we demonstrate that fDEP can be used to mix the contents of the two outer fluids with the central fluid. When an AC electric field is applied across the three streams, the two fluid interfaces polarize and deflect, and forcibly inject their contents into the center stream (Figure 2). This injection rate can be exploited to control the concentration in a downstream microfluidic system.

### 2.1. Device Design and Fabrication

Mixing experiments were performed using a microfluidic channel network with embedded planar electrodes, as shown in Figure 1. Each device was fabricated using a combination of standard soft lithography and microfabrication techniques. Briefly, microchannel electrodes were fabricated using wet chemical etching. Glass cover slips (50 × 30 mm—#1, Fisher Scientific, Hampton, NH, USA) were first coated with 2 nm of chrome and 50 nm of gold using electron beam evaporation. The cover slips were then patterned with photoresist (Shipley 1813) and exposed metal film was etched away with gold and chrome etchant to create an array of metal electrodes. The polymer microfluidic device, which sits atop the glass coverslip was fabricated in PDMS (Momentive, Freemont, CA, USA, RTV 615A). Channels were fabricated in PDMS using a polymer mold constructed out of SU-8 photoresist (Microchem, Westborough, MA, USA). SU-8 was lithographically fabricated onto a silicon wafer using contact lithography. The polymer mold consisted of two different layers—one 3 μm layer and a second 65 μm-tall layer. The 3 μm layer was used to form an array of gradient channels while the 65 μm layer formed the upstream mixing and fluidic routing channels and gradient chambers. First a thin 3 μm layer of SU-8 was patterned onto a silicon water. The layer was patterned using contact lithography and developed. Next, a second 65 μm layer of SU-8 was spin coated onto the wafer and patterned and developed, to produce a dual-layer device. A 1:10 mixture of PDMS elastomer and curing agent was poured atop the SU-8 polymer mold, cured and gently peeled off. Fluid ports were punched into the PDMS using a 0.75 mm biopsy punch (Ted Pella, Inc., Redding, CA, USA). The microchannel and coverslip were exposed to oxygen plasma (Jelight, Irvine, CA, USA, Model 42A) and immediately aligned and sealed under an inverted microscope. The completed device consisted of a network of flow channels 100 μm wide and 65 μm high connected to a series of perpendicular downstream gradient channels. Point electrodes were embedded in the main upstream channel. They were axially separated by 20 μm and symmetrically bridged the channel width. The downstream gradient chamber consisted of two reservoirs, one with an inlet stream consisting entirely of a low conductive buffer. The adjacent reservoir was fed liquid from the upstream, main flow channel. Electrodes with sharp points were utilized in order to maximize the electric field strength across the liquid interface, as the sharp point serves to focus the electric field to the tip of the electrode.

### 2.2. Electrokinetic Flow

AC electric fields have long been exploited to manipulate the fluidic contents of microfluidic systems. Fluid motion is produced through an interaction between an applied electric field and interfacial charge, and when an electric field is applied to a charged surface or liquid domain, an electrical stress is produced that can be used to drive fluid flow. In liquids, these electrokinetic flows occur when a field is applied tangential to polarized surface (e.g., electro-osmosis) or across regions of fluid with spatial gradients in electrical properties (e.g., electrothermal flow). Recently, a new type of electrokinetic flow was discovered in the vicinity of laminar liquid interfaces formed between two miscible aqueous liquids [26]. The phenomenon is known as fluidic dielectrophoresis (fDEP), which describes the frequency-dependent fluid motion of a laminar liquid interface in response to an AC electric field. Interfacial fluid motion is produced using a series of parallel electrodes integrated into a microfluidic t-channel device with three co-flowing liquids (Figure 2a). Each liquid has a different electrical conductivity and dielectric constant such that a large electrical mismatch exists at the interface formed between the two liquids. When an AC field is delivered across the three co-flowing streams, the two fluid interfaces polarize and displace into the center of the microchannel (Figure 2b). The displacement is driven by conductive and dielectric charging (e.g., polarization) at each fluid interface, and is dependent on the electrical properties of each fluid stream and the AC electric field frequency. For a liquid interface subjected to a time varying monochromatic electric field, the displacement is directly proportional to the real part of the interface polarizability factor, K(ω), which is function of field frequency (ω), electrical conductivity and permittivity:

(1) $$Re\left[K\left(\omega \right)\right]=\frac{\left(\varepsilon_{2}-\varepsilon_{1}\right)\tau^{2}\omega^{2}}{\left(\varepsilon_{2}+\varepsilon_{1}\right)\left(\tau^{2}\omega^{2}+1\right)}+\frac{\left(\sigma_{2}-\sigma_{1}\right)\tau^{2}\omega^{2}}{\left(\sigma_{2}+\sigma_{1}\right)\left(\tau^{2}\omega^{2}+1\right)}$$

where $\tau =\left(\frac{\varepsilon_{2}\;+\;\varepsilon_{1}}{\sigma_{2}\;+\;\sigma_{1}}\right)$ is the characteristic Maxwell-Wagner charge relaxation timescale at the interface between the two liquids. fDEP offers a useful method for initiating fluid motion because interface displacement behavior is very sensitive to the relative conductive and dielectric differences across the interface. At frequencies on the order of 100 kHz, for example, the interface displacement is governed solely by differences in the electrical conductivity at interfaces between two co-flowing fluids. At high frequency (typically >10 MHz), however, the displacement is driven by differences in permittivity. fDEP naturally compliments on-chip mixing because these processes often require the combination of two or more fluids with different electrical properties. In the next section of this work we discuss how these fluid properties can be controlled to produce an fDEP response.

### 2.3. Chemicals and Reagents

Fluid motion by fDEP requires a polarizable liquid interface. In this work, liquid interfaces were composed of three fluids with different electrical conductivity (σ) and dielectric constant (𝜀). When forced to flow side-by-side at low Reynolds number these three fluids formed two interfaces, each with a large electrical mismatch. Each stream was injected at a constant flow rate (10 μL/min) into the device using a low-cost flow controller equipped with an externally pressurized fluid-filled cryogenic vial [27]. We labelled each fluid with a different Alexa Fluor fluorescent dye to accurately image the interface position using confocal microscopy. The electrical interface was formed by flowing two outer (green) diluted phosphate buffered saline streams ($\sigma_{1}=1.60\;\mathrm{mS}/\mathrm{cm}$; $\varepsilon_{1}=78$) with 10 ng/mL of Alexa Fluor 488 (Invitrogen, Carlsbad, CA, USA). The center (red) high dielectric stream ($\sigma_{2}=19\;\mu S/\mathrm{cm}$; $\varepsilon_{2}=110$) was comprised of 0.8 M 6-aminohexanoic acid (Sigma-Aldrich, St. Louis, MO, USA) (AHA) labeled with 10 ng/mL of Alexa Fluor 594 (Invitrogen). AHA is a water-soluble zwitterion used for increasing the dielectric constant of aqueous solution. Prior to fluorescent labeling, the AHA solution was polished with 1 g/mL Dowex MR-3 (Sigma-Aldrich) ion exchange resin to remove trace salts and reduce solution conductivity.

### 2.4. Characterizing the Magnitude of Mixing

**Figure 3:**
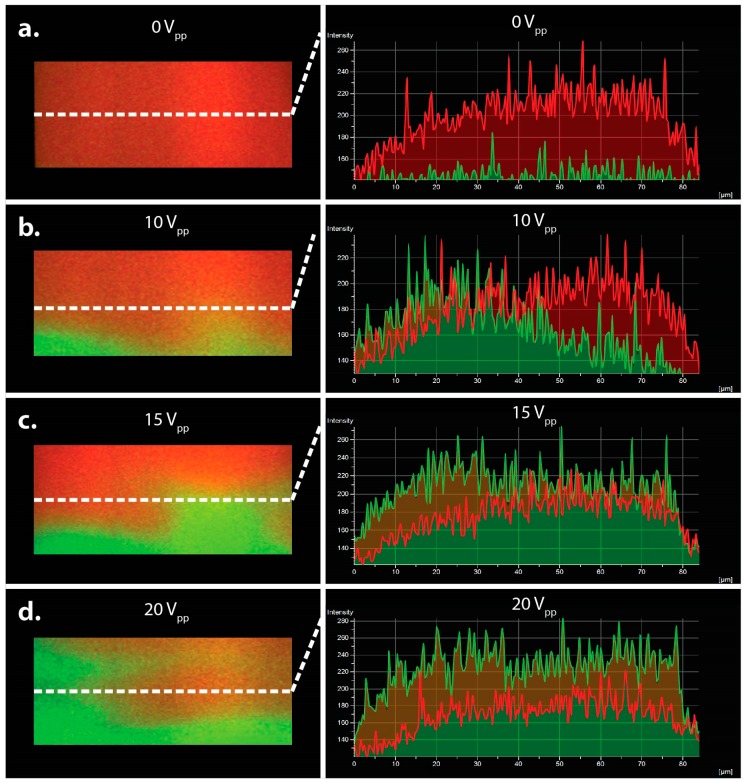
3D cross-sectional image of the device at the entrance of the gradient chamber. Fluorescent profiles (**a**) 0 Vpp; (**b**) 10 Vpp; (**c**) 15 Vpp and (**d**) 20 Vpp is applied across the tri-laminar flow streams The fluorescent measurements were taken in center of the microchannel cross-section and indicated by the dotted white line.

To analyze the degree of mixing and the voltage dependence, measurements were taken over a 100 × 100 square μm section directly above the electrode array. Interfacial motion was captured using a confocal microscope (Nikon/Prairie Technologies) equipped with an Andor iXon 897 camera, two 50 mW solid-state lasers for excitation at 488 nm and 561 nm, respectively, and a triggered Piezo Z stage for capturing 3-dimensional micrographs of the microchannel cross-section. When fDEP was used to drive fluid motion, the resulting mixing was calculated by capturing a fluorescent intensity profile over the microchannel cross section depicted in Figure 3. Using these confocal images, the intensity profile in the central fluidic stream was analyzed approximately 30 μm above the channel surface across the entire channel width. Each intensity profile was normalized to the fluorescent background (~126 intensity units) when no AC electric field was applied (Figure 3a). Mixing was then quantified by averaging the fluorescent intensity of the Alexa Fluor 488 across the channel width, normalizing this value with the background intensity profile and then dividing by the theoretical max intensity.

## 3. Results and Discussion

### 3.1. Mixing with Fluidic Dielectrophoresis

**Figure 4:**
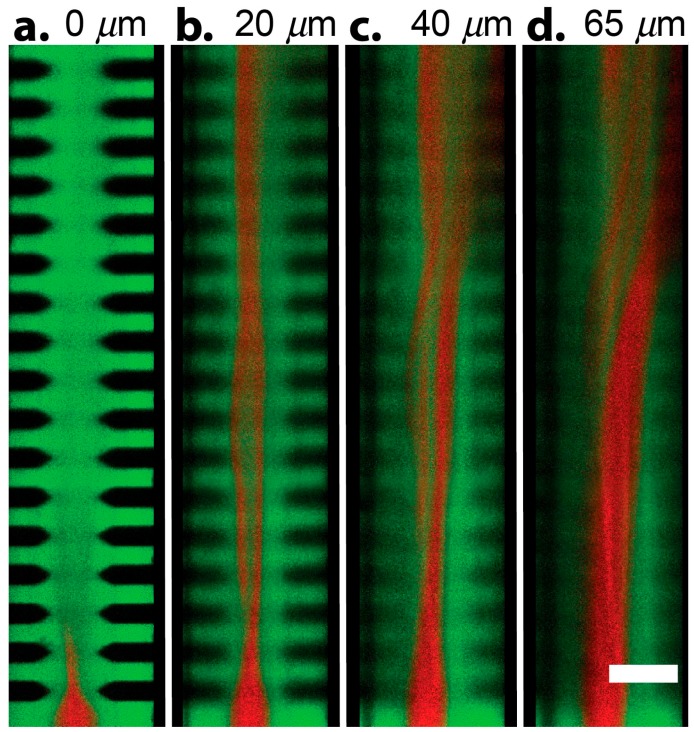
A confocal montage captures fluid motion down the axial length of the mixing channel captured at different positions along the channel height. (**a**) Bottom surface of the microfluidic device. Upon entering the array with an applied voltage of 20 MHz at 1 MHz, the outer green streams inject into the center occupying the entire bottom of the channel; (**b**) 1/3 from the bottom of the device; (**c**) 2/3 from the bottom of the device; (**d**) Top of the microchannel device. Scale bar, 50 μm.

We first sought to investigate the ability to mix multiple fluidic streams using fDEP. We observed rapid mixing between three co-flowing fluid streams when an AC voltage was applied across the microchannel. Mixing was observed to occur when the high conductive (green) stream forcibly displaced into the low conductive (red) stream. In order to better investigate the mechanism by which mixing occurs, confocal images of the channel cross-section were taken for three different voltages—10, 15 and 20 V_pp_. The degree of mixing between the red and green streams was quantified using fluorescent intensity profiles captured over the microchannel cross-section (Figure 3). Shown in Figure 3, mixing occurs when the central green stream is forcibly displaced into the low conductive red stream. When the applied voltage was sufficiently large the rate of injection is so great that an electrokinetic vortical flow formed down the axis of the mixing electrode array, as shown in Figure 4. Therefore, fDEP served to electrokinetically inject fluid from the outer two flow streams and to satisfy conservation of mass, fluid volume from the central stream was dispelled outward to the two outer streams. Therefore, the electrokinetic mixer offers a means to mix with less dilution since newly injected flow of the outer fluid is accompanied by an equal outward flow of the inner fluid.

### 3.2. The Influence of Field Frequency and Voltage on Mixing

**Figure 5:**
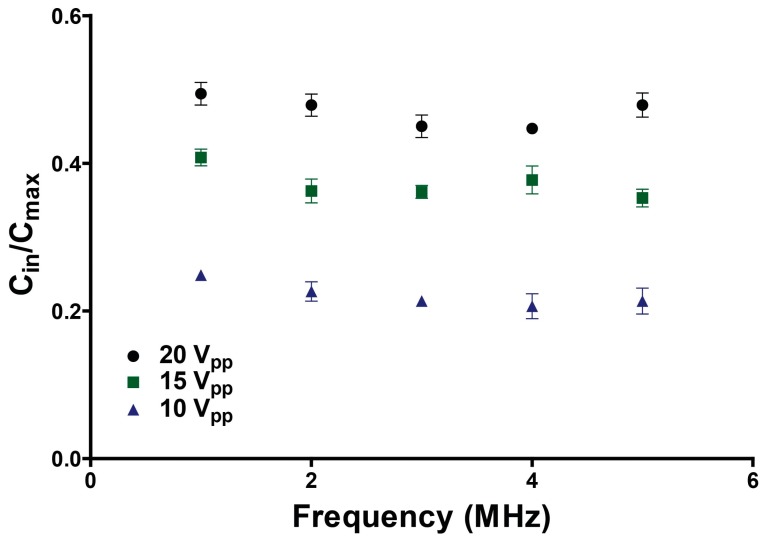
Quantification of the degree of concentration perturbation (C_in_/C_max_) versus frequency. The concentration is normalized based on the theoretical max concentration.

**Figure 6:**
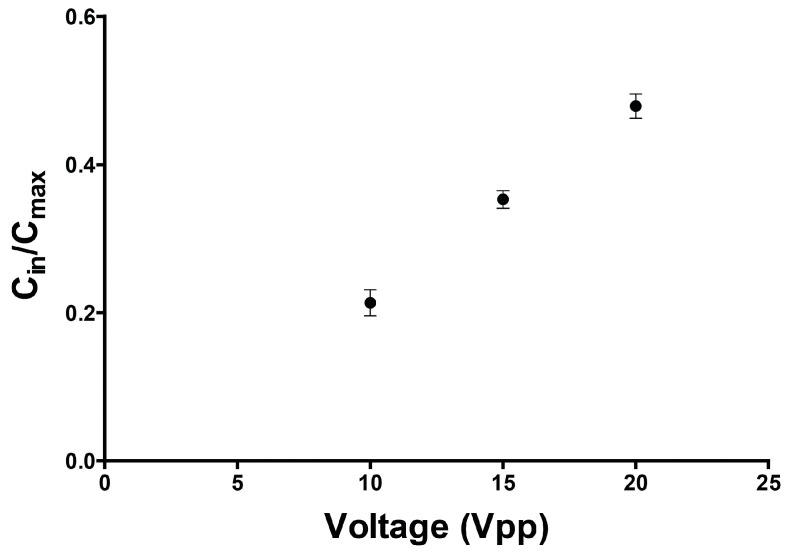
Plot of C_in_/C_max_ versus applied voltage. The concentration of solute increases linearly with increasing applied voltage.

There are several important properties of the AC electric field that will influence fluid mixing. The field frequency will affect the direction of interfacial displacement. A low frequency field, for example, will force high conductive fluid from the two outer streams to displace into the central low conductive stream. Alternatively, if a high frequency electric field is applied across the fluid interfaces the center high dielectric stream displaces into the two adjacent low dielectric streams. In this case, no mixing was observed. Thus, for this system where we sought to inject solute from the two outer flows, the frequency regime significant for the experiments was at low frequency (<5 MHz). The influence of AC frequency on mixing is shown in Figure 5. For the frequency ranges from 1 to 5 MHz the average normalized concentration of the high conductive stream is not significantly influenced by the AC field frequency. The observed solute concentration entering the gradient chamber (C_in_) was normalized by the theoretical maximum concentration. However, it was apparent that the applied voltage impacted the mixing concentration. Therefore, we next investigated the effects of applied voltage on mixing. To do this, three different AC voltages—10, 15 and 20 V_pp_—were applied across the fluid interfaces at a constant field frequency of 5 MHz. While we could have selected any frequency between our 1–5 MHz experimental range based on the data in Figure 5, we based our concentration data on the largest experimental frequency simply because higher frequency electric fields are known to be less likely to produce Faradaic reactions and Joule heating in conductive buffers. Shown in Figure 6, we observed a linear correlation between the applied voltage and degree of mixing. In particular, as the applied voltage increases, the degree of mixing also increases.

### 3.3. Using AC Electrokinetic Mixing to Create Finely Tunable Concentration Gradients

**Figure 7:**
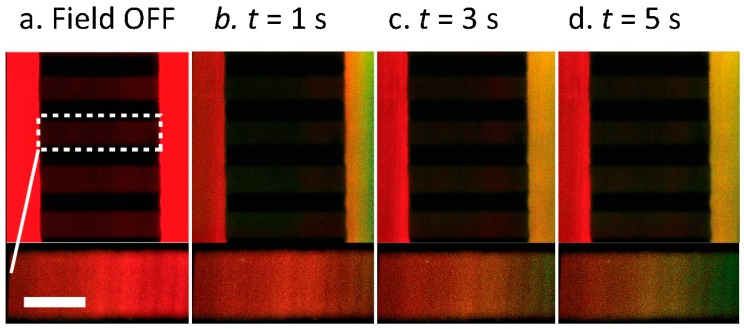
Magnified view of the microfluidic gradient chamber. Images were captured at varying time points after the electric field was applied over a period of 5 s. When a 5 MHz, 20 V_pp_ AC electric field is applied upstream it modifies the concentration of mixed solution that enters the gradient chamber. (**a**) When the electric field is off, the connecting gradient side channel lacks any fluid from the tri-laminar outer streams. However, when the field is switched on a chemical gradient forms across the connecting channel, which increases with over a time period of 5 s; (**b**) 1 s; (**c**) 3 s and (**d**) 5 s. Scale bar, 40 μm.

Finally, with the ability to induce mixing and control the solute concentration within the central stream, we investigated the ability to integrate the upstream electrokinetic mixer with a downstream concentration gradient generator. Shown in Figure 2a, when no AC electric field is applied, no fluid injection occurs and the flow central fluid stream, which is fed into the downstream chamber consists entirely of the low conductive fluid (red stream). However, when an AC electric field was applied upstream, fluidic mixing occurred and altered the downstream fluidic concentration. Figure 7 shows the gradient chamber when an upstream AC electric field of 5 MHz, 20 V_pp_ was applied. A series of consecutive micrograph images were captured at different time intervals to illustrate the rate at which the gradient is generated when upstream mixing is initiated. After the electric field was activated, a concentration gradient is formed within the gradient channels, where one chamber consists entirely of the low conductive (red) buffer and the adjacent stream consists of a mixture of the high conductive (green) and low conductive (red) buffers. When differing voltages are applied, the time for gradient generation, as well as the gradient steepness differ due to differing magnitudes of mixing and different rates of fluid injection. With lower applied voltages, less mixing occurs which produces a lower concentration gradient. Larger applied voltages result in a greater rate of fluidic injection and produce a steeper gradient.

## 4. Conclusions

In this paper, we have presented a novel method for electrokinetic mixing in a microfluidic device using fluidic dielectrophoresis. Using an AC electric field, the two laminar liquid interfaces between three co-flowing fluids were actively mixed through the use of interfacial electrokinetic stresses. When an electric field was applied across these interfaces, they polarized and forcibly injected the contents of the two outer streams into the center stream. The degree of mixing was monitored by labeling each fluid stream and measuring the fluorescent intensity profiles over the microfluidic channel cross-section. Mixing was shown to be influenced by both the frequency and voltage of the applied AC electric field. As the voltage increased, the magnitude of mixing increased. It was shown that there exists a linear relationship between the degree of mixing and applied voltage. Finally, we coupled this upstream mixing device with a downstream passive gradient chamber to demonstrate the usefulness of this proposed method. Upstream, an AC electric field was used to induce mixing. This led to a change in solute concentration that was sent downstream to the gradient chamber. By controlling the degree of mixing, we can alter the steepness of the concentration gradient. Future work will focus on applying this novel electrokinetic mixing component in order to create tunable on-chip concentrations.
